# Supplementary material for: Are carbon emissions trading and green financial instruments synergistic? -Comprehensive quantitative research based on content analysis
Source: PLoS One. 2024 Mar 7;19(3):e0298601. doi: 10.1371/journal.pone.0298601 (PMC10919601; doi:10.1371/journal.pone.0298601)
Supplement: S1 Data — (ZIP) [file pone.0298601.s001.zip › Data/地级市Z_F工作报告.zip!/╦─┤¿/─┌╜¡╩╨/└┤╫╘╣1⁄2╓┌║┼í╛┬φ┐╦╩2╛▌═°í┐_2015.docx]

各位代表：

　　我代表市人民政府，向大会报告工作，请予审查，并请市政协委员提出意见。

2014年工作回顾

　　2014年，在省委、省政府和市委的坚强领导下，在市人大、市政协的监督支持下，市政府全面贯彻落实中央、省、市各项决策部署，牢牢把握科学发展、加快发展的工作指导思想，深入实施“三大发展战略”，突出抓好“七项重点工作”，把稳增长作为首要任务，同步促改革、调结构、惠民生、防风险，全市经济发展稳中有进、稳中提质，各项事业全面进步，社会大局和谐稳定，就业形势和物价水平总体平稳，人民群众安居乐业。全市实现地区生产总值（GDP）1156.77亿元，增长8.9%；全社会固定资产投资697.77亿元，增长21.2%；地方一般公共预算收入45.08亿元，增长16.6%，地方一般公共预算收入占GDP比重提高0.37个百分点，税收收入占地方一般公共预算收入比重提高0.4个百分点；城镇居民人均可支配收入23162元，增长9.7%；农民人均纯收入9565元，增长11.4%。

　　一、深入实施“两化”互动、城乡统筹战略，“四化”同步进程加快

　　新型工业化平稳推进。加快老工业基地调整改造，实现工业总产值1760.95亿元，增长7.2%；工业入库税金24.32亿元；规模工业增加值增长9.4%；全市完成工业技改投资202亿元。新增规模工业企业28户、5亿元企业20户、10亿元企业6户。七大工业园区新增承载面积5.1平方公里，新入驻单项投资5000万元以上企业（项目）38个，实现主营业务收入1635.8亿元，增长14.4%。强化节能降耗，关闭、淘汰落后产能企业28户，关闭小煤矿12户，预计万元GDP综合能耗下降5.0%。

　　信息化水平不断提升。成功进入“国家信息惠民试点城市”，数字城管、数字地理信息等6个智慧城市重点项目相继启动。加快城市新一代通信基础设施建设，新建4G基站1943个，城区光缆覆盖率95%。推进“宽带乡村”建设，新增通宽带行政村540个，各县（区）均进入全国或全省“宽带乡村”试点。工业化与信息化加快融合，新增两化融合示范带动企业30户，实施信息化技术改造传统产业项目15个。

　　新型城镇化步伐加快。新增城镇建成区面积11平方公里，新增城镇人口6.09万人，城镇化率提高1.54个百分点，达44.21%。内江主城区面积70平方公里，常住人口71.4万人。加快建设公共基础设施和服务设施，加快建设公园绿地和生态系统，大千路北延线、北环线三四段等10条道路全面竣工，市体育中心、市文化中心等9个公共服务项目前期工作进展有序，塔山公园生态复合绿道、小青龙河景观绿道等4个生态绿地项目快速推进，甜城湖左岸（师院西区至二医院段）生态廊道全面建成；城南新区、白马组团、椑木组团加快建设。积极夯实底部基础，资中沱江北岸古城保护、南岸新区开发加速实施，隆昌城中有湖、湖中有城、景城一体格局加速构建，威远城市综合体、婆城公园、威远河景观建设加速推进，县城控规覆盖率均在85%以上。“百镇建设行动”深入推进，11个镇入选全国重点镇。围绕“彻底治脏、大力治乱、全面治差”总要求，集中开展市容秩序、环境卫生、交通秩序、建设工程扬尘、油（煤）烟和噪声污染等六类综合整治，市容市貌明显改善。启动“交通治堵三年攻坚战”，完成省道206线与双洞路平交道口改造，叠园路等一批治堵项目正抓紧实施，沱江大桥复线桥、邱家嘴立交桥、桐梓坝大桥南成渝铁路桥下道路拓宽改造等项目前期工作加快推进。城市过境高速公路开工，成渝客专（内江段）、内威荣高速公路（内江段）、自隆高速公路（内江段）、花园滩大桥建设加快，新入城线玉王庙互通立交枢纽工程竣工通车，城市轨道交通完成项目论证和规划编制。

　　农业现代化稳中有升。创建国家农业科技园区通过评审。稳定粮食生产，粮经复合型现代农业产业基地、现代农业产业万亩示范区、省级农业标准化示范区建设取得实效，新建高标准农田8.36万亩，粮食产量151.23万吨。现代畜牧业试点市建设深入推进，规模养殖比重逐步提升。注重产业化主体培育，新登记家庭农场468家，新增省级示范农民合作组织6个、省级农业产业化龙头企业3个。强化农产品质量安全，新通过无公害产品认证41个，绿色食品认证11个，内江获“中国黑猪（内江猪）之乡”、威远县获“中国无花果之乡”称号。狠抓基础设施建设，向家坝灌区一期工程通过国家发改委咨询评估，联合水库开工建设，象鼻咀水库建成投入使用，两河口水库前期工作加快推进，新（改）建农村公路1536公里，新建农村户用沼气池6262口，完成农村电网升级改造工程485个。威远县、隆昌县“省级新农村建设成片推进示范县”建设扎实有效，全市4个新农村综合体加快建设，新建成幸福美丽新村40个、新村聚居点123个。

　　二、深入实施创新驱动战略，转型升级步伐加快

　　高新区建设取得突破。将高新区建设作为全市“一号工程”，统筹推进“一区三园”建设，成功创建省级高新技术产业园区。完成高新区总规、控制性详规及产业发展规划；引进高新技术产业项目3个，协议投资28亿元；入驻科研团队、博士工作站、企业技术中心33个；实施园区道路、安置还房、污水处理厂等项目37个。隆昌园区建成省级科技企业孵化器，白马园区1万平方米、高桥园区5000平方米科技企业孵化器建设步伐加快。

　　自主创新能力不断增强。发挥企业主体作用，新建省级工程技术研究中心1个、省级企业技术中心4个、市级企业技术中心28个，组织实施重大科技成果转化项目42项。新增专利申请660件，新增中国驰名商标1枚、四川省著名商标5枚。实施质量强市战略，新创四川名牌产品18个，内江主导编制的《循环流化床锅炉砌筑工艺导则》上升为行业标准。强化人才支撑，培养、引进各类紧缺和高层次人才884名。

　　新兴产业快速发展。页岩气产业培育加速，威远区块首条外输干线建成投运，域内建成钻井平台12个、开钻气井45口、日产气20万立方米，年度投资14.5亿元。信息安全产业活力初显，数据恢复省级重点实验室通过验收，信息安全产业孵化园加快建设。电子信息产业高速发展，实现产值25.26亿元，增长55.0%。节能环保产业发展势头强劲，生产经营企业达66家，主营业务收入100亿元。大力发展高新技术产业和战略性新兴产业，新认定国家高新技术企业21家、市级以上高新技术产品30个、市级以上战略性新兴产品24个，高新技术产业、战略性新兴产业产值分别增长44.0%、22.0%，占规模工业总产值比重分别提高4.6、6.9个百分点。

　　现代服务业蓬勃发展。把发展电子商务和现代物流业作为发展现代服务业的“一号工程”，全力促进服务业发展提速、比重提高、水平提升。成功举办“四川电子商务暨现代物流发展高峰论坛”和“大千网交会”，加快建设西部电子商务中心、川南电子商务城等项目，启动建设天虎云商川南运营中心、四川农产品数据化智能供应链等电商交易平台，威远连界钢铁物流中心建成投运。全年网络交易额居全省第3位，达363亿元，增长21.0%。启动旅游“138”计划，加快实施一批旅游重点项目，成功创建“四川省旅游标准化示范市”，实现旅游总收入136.97亿元，增长24.1%。内江国际会展中心建成投入使用，一批城市商业设施建成并营业，城乡消费进一步扩大，社会消费品零售总额356.22亿元，增长12.7%。新引进域外金融机构2家、准金融机构6家，1户企业登陆全国中小企业股份转让系统。银行业金融机构本外币各项存款余额1136.16亿元,比年初增加56.69亿元，增长5.3%；贷款余额610.14亿元，比年初增加95.79亿元，增长18.6%。第三产业增加值实现258.34亿元，增长9.9%。

　　三、深入实施改革开放战略，发展活力明显增强

　　各项改革稳妥推进。农村产权制度改革走在全省前列， 46个乡镇、676个村完成产改任务，流转土地47.52万亩，市中区纳入全国第二批农村改革试验区。农村信用社改制工作迈出新步伐，内江市农村商业银行组建获省政府批复，威远县农村商业银行正式挂牌。行政审批制度改革深入推进，取消、下放、调整行政审批事项288项，保留市级行政审批事项84项，行政审批效率综合提速78%。新一轮政府机构改革任务基本完成，事业单位分类改革工作取得阶段性成果。理顺市、区两级城市管理体制，城市管理水平全面提升。工商登记制度改革成效明显，新登记企业1856户，增长72.2%，民营经济活力进一步释放。探索土地集约节约利用新路，成为全国第二批工矿废弃地复垦利用试点市。推进财政预算改革，加强财政绩效管理，盘活财政存量资金2.58亿元。深化医疗卫生体制改革，县级公立医院药品加成全面取消，分级诊疗工作扎实开展。市电影公司、市广电网络公司转企经营和市川剧团体制改革顺利推进。

　　开放合作成效显著。大力实施“高新产业引进、知名企业引进、人才带项目引进”三大攻坚战，成功举办“欧洽会﹒内江专场推介会”等一系列开放活动，新引进高新技术产业和战略性新兴产业项目76个，世界500强企业投资项目10个、中国500强企业投资项目13个，人才带项目11个。全市实际到位市外资金415.02亿元，同口径增长17.9%，其中制造业到位资金282.89亿元，占到位市外资金的68.2%。区域合作、校地合作不断加强，与西南大学、电子科大等5所大学签订合作协议。内江海关获批设立，全年实现外贸进出口总额3.15亿美元。

　　四、全力保障和改善民生，发展成果持续惠民

　　民生实事全面完成。“十项民生工程”、20件民生实事等民生支出完成124.84亿元，占一般公共预算支出的70.7%，提高1.8个百分点。新增城镇就业5.1万人，城镇登记失业率控制在3.99%。完成扶贫投资8000万元，帮助6.83万农村贫困人口脱贫解困。为城乡困难群众安排医疗救助资金8935万元，城乡低保月人均补助标准分别提高至236元和108元。城乡养老保险实现并轨，建成城乡社区日间照料中心79个，为80岁以上和困难家庭失能人员共5.33万人提供了居家养老“五助”服务。全面建立城乡居民大病保险制度，城镇居民基本医疗保险覆盖52.4万人，新农合参合率99.8%。创新推进棚改工作，完成投资39.1亿元，改造城镇危旧房和棚户区14047户；实施农村危房改造7342户；建设保障性住房6469套；做好失地农民安置还房建设工作，在保证不欠新账的前提下，所有安置还房项目全面开工。解决18.4万农村人口和3.85万学校师生安全饮水问题，开展覆盖全市的农村水井水质监测工作。提升乡村公路通达能力和安全水平，新建公路安全护栏827公里，基本实现村村通油路（水泥路）。市场物价平稳，居民消费价格上涨1.4%。

　　社会事业协调发展。学前三年毛入学率87.2%，小学、初中入学率分别为100%、98.4%，普通高中教育质量名列全省前茅，藏区“9+3”中职免费教育毕业生100%就业，川南幼专创建成功，内江师院整体搬迁、内江六中高新校区建设、四川健康职业学院创建等工作相继启动。市一医院、二医院、中医院新区项目加快推进，完成乡镇卫生院建设项目25个，新建村卫生室40个。范长江故居（纪念馆）改陈布展重新开放，大千园整体改造提升等项目开工；“资中木偶戏”列入国家级非物质文化遗产保护名录，市中区获“中国民间文化艺术之乡”称号，话剧《范长江》获省第三届“文华奖”。全民健身运动蓬勃开展，参加第十二届省运会取得金牌数、总分数全省第三的好成绩。落实“单独两孩”政策，人口自然增长率4.16‰。

　　生态建设得到加强。城市集中式饮用水水源地水质达标率100%，沱江内江段长期保持Ⅲ类水质。完成内江第一污水处理厂改建工程，城区污水集中处理率88%，生活垃圾无害化处理率99%。综合治理河道21公里，甜城湖清淤疏浚工程全面完成，威远河、隆昌河水质得到改善。开展涉重金属企业强制性清洁生产审核，主要污染物总量减排任务顺利完成。加强空气质量监测和大气污染防治，淘汰黄标车和老旧车4030辆。“绿色中国行”大型公益活动首次入川走进内江，启动省级森林城市创建工作，建成生态乡镇7个、生态村10个、生态家园100户，全市森林覆盖率32.56%。

　　社会保持安全稳定。加强安全生产能力建设，市级安全生产综合监控指挥中心投入使用，建成省级安全社区9个、全国安全社区3个；狠抓煤矿安全生产工作，开展“道路交通安全综合整治攻坚年”行动，加强消防、危化品、食品药品等重点行业领域安全生产监管，各类生产经营性安全事故起数、受伤人数同比分别下降19%、21%，死亡人数、直接经济损失同比持平，未发生重大及以上安全事故。社会管理网格化服务体系全面建成，群众来信来访得到有效处理，“大调解”工作全面深化，成功调解处置各类矛盾纠纷853件。“平安内江”建设持续推进，立体化治安防控体系加快构建，公众安全感满意率95.2%。应急处置工作不断加强，各类突发事件得到及时妥善处置。

　　国防动员、人民防空、民兵预备役建设和双拥优抚等工作不断加强，军政军民关系更加巩固。第三次全国经济普查工作如期完成。机构编制、统计、调查、气象、水文、防灾减灾、人防、检验检疫、无线电管理、保密、档案、史志、外事侨务、民族宗教等工作迈出新步伐，红十字、慈善、老龄、广播电视、妇女儿童、残疾人等事业取得新成绩。

　　五、认真开展党的群众路线教育实践活动，政府自身建设不断加强

　　工作作风明显转变。按照“照镜子、正衣冠、洗洗澡、治治病”的总要求，扎实开展党的群众路线教育实践活动。深入群众实施定点帮扶，着力打通服务群众“最后一公里”，认真开展批评与自我批评，扎实推进正风肃纪，先后“废、改、立”相关制度26项。深入开展“庸懒散浮拖”专项整治，积极开展党风政风行风群众满意度社会评价，畅通效能监督渠道。严格执行中央“八项规定”、省市“十项规定”，转变文风会风，强化预算约束，大兴节俭之风，全市“三公”经费下降17.8%。

　　依法行政扎实推进。坚持重大事项向人大及其常委会报告、规范性文件向人大常委会备案，自觉接受人大及其常委会的法律监督、工作监督；坚持协商在前，自觉接受政协的民主监督，广泛听取民主党派、工商联、无党派人士、人民团体的意见；积极支持工会、共青团、妇联等群团组织开展工作。办理人大代表议案4件、建议107件，满意率100%；办理政协委员提案312件，满意率99.7%。注重法制宣传教育培训，坚持政府常务会集中学法制度，公务人员依法行政意识进一步增强。完善法律顾问制度，扎实开展专家论证、技术咨询、决策评估、合法性审查、民主协商、公示听证，依法决策、科学决策水平有力提升。规范行政处罚裁量权，强化行政复议工作，努力保障人民合法权益。深化政务公开，市、县行政权力公开运行平台实现全覆盖，公开市级行政职权事项3896项。

　　廉政建设全面加强。坚持“一岗双责”，严格落实党风廉政建设责任制，惩治和预防腐败体系进一步巩固。充分发挥财政、审计、监察职能，认真组织开展领导干部和国有企业负责人经济责任审计工作，加大对惠民资金、征地拆迁、民生项目等重点领域和关键环节的监管力度，完成政府投资工程预决算审计项目537个，核减资金3.03亿元。查处一批群众反映强烈的突出问题，政纪立案82件，政纪处分85人，强化了在监督下工作的风气和机制。

　　各位代表，过去一年，遇到的困难比预料的多，取得的结果比预料的好。面对经济下行压力，我们突出发展第一要务，出台十八条措施稳定经济增长，保持了高于全国全省平均水平的发展速度；我们加快转方式、调结构、促升级，大力发展高新技术产业和战略性新兴产业，经济结构出现可喜变化；我们以经济体制改革为重点，统筹推进各领域改革，全面深化改革实现扎实开局；我们全力保障和改善民生，人民生活水平不断提高。这些成绩的取得，是市委统揽全局、科学决策的结果，是市人大、市政协监督支持、共谋发展的结果，是全市人民同心同德、顽强拼搏的结果！在此，我谨代表市人民政府，向430万内江人民，向人大代表、政协委员、各民主党派、工商联、无党派人士、各人民团体，向驻内部队、武警官兵、政法干警和民兵预备役人员，向关心、支持和参与内江建设发展的各界朋友，表示衷心感谢并致以崇高敬意！

　　在看到成绩的同时,我们更加清醒地认识到,经济社会发展还存在一些困难和问题。受宏观经济下行等因素影响，地区生产总值、规模工业增加值、服务业增加值、社会消费品零售总额、城镇居民人均可支配收入、城镇化率等6项指标增速虽然完成了省下达的任务，但未达到我市年初确定的预期目标；传统产业占比较大，高新技术产业和战略性新兴产业尚未形成规模，新的增长动力不足，转方式、调结构任务艰巨；土地、环境空间约束加剧，政府投资能力受限，民间投资增幅下滑，投资增长乏力；财政增收困难，政府性债务以及金融、房地产等领域潜在风险化解压力较大；城市基础设施不完善、功能分区不合理，交通拥堵还未明显缓解；加强水资源管理保护、改善空气质量任重道远；整治“庸懒散浮拖”现象还需加大力度，政府依法行政和反腐倡廉建设还需加强。对这些问题，我们将高度重视，采取有效措施，认真加以解决。

2015年工作安排

　　2015年是全面深化改革的关键之年，是全面推进依法治市的开局之年，也是全面完成“十二五”规划的收官之年。今年政府工作的总体要求是：以邓小平理论、“三个代表”重要思想、科学发展观为指导，认真贯彻落实习近平总书记系列重要讲话精神，全面贯彻党的十八大、十八届三中、四中全会及省委十届四次、五次全会精神，按照市委六届八次、九次全会的总体部署，主动适应经济发展新常态，坚持科学发展、加快发展工作指导思想，坚持稳中求进工作总基调，深入实施“三大发展战略”，着力稳增长、促改革、调结构、惠民生、防风险，提升发展质量，增进民生福祉，推进依法治市，维护社会稳定，努力建设充满希望、充满活力的幸福美丽内江。

　　今年全市经济社会发展的主要预期目标建议：地区生产总值增长8.0%左右，其中第一产业增加值增长3.6%、第二产业增加值增长8.4%（其中规模工业增加值增长8.0%）、第三产业增加值增长9.8%；地方一般公共预算收入增长10.0%；社会消费品零售总额增长11.8%；全社会固定资产投资增长14.7%；城镇居民人均可支配收入增长9.2%，农民人均纯收入增长10.1%；居民消费价格涨幅控制在3.0%以内；城镇登记失业率控制在4.5%以内；人口自然增长率控制在5.4‰以内；完成省下达的节能减排目标任务。

　　需要报告的是，在社会主义市场经济条件下，经济增长目标是预期性的弹性指标，且主要依靠市场主体自主行为去实现。8.0%左右是一个区间值，充分考虑了需要和可能。研究提出今年经济社会发展预期目标，我们充分考虑了国际、国内经济发展大势和我市现阶段发展的客观要求。当前，世界经济深度调整、复苏艰难，我国经济发展进入以速度变化、结构优化、动力转换为主要特征的新常态，正处于爬坡过坎、攻坚克难的关键时期，经济下行压力依然很大，面临的困难可能更多。内江与全国、全省一样，经济正在向形态更高级、分工更复杂、结构更合理的阶段演化，发展速度正从高速增长转向中高速增长，发展方式正从规模速度型粗放增长转向质量效率型集约增长，经济结构正从增量扩能为主转向盘活存量、做优增量并举的深度调整，经济发展动力正从传统增长点转向新的增长点。作为老工业城市，内江传统产业、过剩产能占比高的现实没有改变，新常态带来的影响可能会更广泛、更深远、更持久；作为西部内陆地区，内江“底子薄、不协调、欠发达”的基本市情没有改变，全市人民期盼加快发展的愿望十分强烈。在这种背景下，我们必须“抓紧转”，主动出击促转型，向改革创新要动力、要活力，向转方式调结构要质量、要效益，推动经济从要素驱动、投资驱动向创新驱动转变，从高速增长向高效增长转变；必须“加快赶”，专注定力稳增长，高度重视、大力支持实体经济发展，主动作为、奋发有为，保持稳增长与调结构、防风险之间的平衡，确保经济运行处于合理区间，实现更高水平、更好质量、更加协调的发展。

　　围绕上述总体要求和预期目标，重点抓好以下工作：

　　一、深化结构调整，促进工业提质增效

　　改造提升传统产业。加快老工业基地调整改造，应用高新技术和先进适用技术改造提升传统产业，完成技改投资200亿元，推动冶金建材向新材料、机械汽配向高端装备制造、医药化工向生物医药产业转型升级，壮大食品饮料产业，稳定电力能源产业。加强对重点工业企业的监测、帮扶，促进企业稳定生产。加快工业企业培育，新增规模工业企业20户以上、新注册私营企业1000户以上。坚持集聚发展，工业园区产业集中度提高1.5个百分点以上，七大工业园区新入驻单项投资5000万元以上企业（项目）20个。

　　发展高端成长型产业。坚持在创新驱动中促转型、在优化结构中稳增长，推动经济发展提质增效升级。突出发展信息技术、新材料等产业，推动产业向中高端水平迈进，高新技术产业、战略性新兴产业产值占规模工业总产值比重分别提高3个、2.5个百分点。大力发展高端成长型产业，加大页岩气就地转化和产业化培育力度；加快建设信息安全产业孵化园，在数据处理、物联网等领域尽快形成有市场竞争力的产品；加快培育节能电力、绿色建材等节能环保产业，推进66个节能环保产业项目建设，力争实现主营业务收入120亿元。

　　提升自主创新能力。继续把高新区建设作为全市“一号工程”，启动国家高新区申报工作，实施园区道路、污水处理厂等20个项目，新增承载面积1平方公里；完成白马园区、高桥园区科技企业孵化器建设，新增入孵企业30户以上。深化产学研合作，推进项目、资金、人才、平台等创新要素向企业集聚，新建一批省级工程技术研究中心、企业技术中心、重点实验室、院士（专家）工作站，组织实施重大科技成果转化项目40项。加强知识产权保护，积极申报专利，新增国家地理标志保护产品1个，新培育四川省著名商标5枚、四川名牌产品10个。提高研发设计、流程控制、管理营销等环节的信息化应用水平，新增两化融合示范带动企业27户。

　　二、发展现代服务业，加快培育经济发展新引擎

　　大力发展电子商务和现代物流业。实施电子商务“1259”发展规划，加强电子商务与实体经济融合。结合国家信息惠民试点城市和智慧城市建设，继续实施“全企上网”、“宽带乡村”工程，抓好国家电子商务进农村综合示范县建设，引导工业企业和农业经营主体发展电子商务，培育省级重点电商企业3户。办好电子商务博览会，加快内江电子商务产业园等项目建设，努力争创全省首批电子商务示范城市。加快一批重点物流项目建设，搭建物流信息平台，完善物流配送体系，积极吸引国内外大型物流企业落户，着力培育AAAA级现代物流企业，促进本土物流企业专业化、规模化发展。

　　加快发展现代旅游业和现代金融业。继续实施旅游“138”计划，推进圣水寺佛教文化旅游区、范长江文化旅游园区等项目建设，完成塔山公园复合绿道、小青龙河景观绿道、石板河旅游景区等3个项目一期工程，加快隆昌古宇湖等创建AAAA级旅游景区工作，全年旅游总收入增长20%以上。鼓励金融机构优化网点布局，开发金融特色产品，扩大金融IC卡在公共服务领域的应用；优化资本市场结构，加强企业上市培育，鼓励企业发行融资债券，大力发展保险服务业；培育发展面向小微企业和“三农”的融资性担保业；依法打击非法集资行为，优化金融生态环境。

　　积极培育科技服务业和养老健康服务业。围绕研发设计、检测检验、技术交易等，搭建科技服务平台，完成大数据中心建设，加快发展科技服务业。坚持多种投资主体共存、多种养老方式互补、多种养老服务组织并举，突出供养、教育、护理、康复一体化经营，完善养老服务体系，推动养老健康与旅游休闲、健康食品等产业融合发展。

　　着力提升传统服务业和扩大消费需求。打造谢家河、高铁片区等现代商圈，提升业态水平，促进服务业与工农业协同发展。加快发展会展业，培育具有内江地域特色的会展品牌，办好迎春购物节、大千美食文化节等展示展销活动。扩大汽车、住房等大宗消费和文化教育、餐饮住宿、体育健身等服务消费规模，努力挖掘农村消费潜力。强化市场监管，打击商业欺诈、制假售假行为，保障市场供应。

　　三、稳定农业生产，促进农村发展农民增收

　　提升产业化水平。严格保护耕地，稳定粮食生产。发挥农业科技园区示范引领作用，带动一批示范基地建设，壮大一批龙头企业，促进农业适度规模经营，延伸城乡之间、工农之间产业链条。做响一批农产品品牌，稳步提升粮油、蔬菜、水果、生猪、家禽产业化水平和市场竞争力。健全农业社会化服务体系，在农机作业、病虫害防治、育种育苗、储藏保鲜等方面推广订单式、托管式服务。扩大特色农业保险试点。加强农产品质量监管。

　　加快新农村建设。按照“业兴、家富、人和、村美”的要求，以“新建、改造、保护”为基本形式，加快推进威远县、隆昌县“省级新农村建设成片推进示范县”项目，建设幸福美丽新村110个、新村聚居点200个，启动农村廉租房建设1000户，加快解决农村危房户、住房困难户的住房问题。加强水资源统筹规划，开工建设两河口水库，加快建设联合水库，抓好石盘滩灌区、龙江水库等大（中）型灌区续建配套项目，完成“十二五”农村饮水安全规划目标任务；建设高标准农田8万亩，新增农村户用沼气池5400口，新（改）建农村公路150公里以上，完成农村电网升级改造工程627个。

　　拓宽农民增收渠道。继续强化农民增收负责制，全面落实强农惠农富农政策，稳步提高农村社会保障水平，增加农民转移性收入。加强农民工职业技能培训，提高工资性收入水平。开发农业多种功能，因地制宜发展高效农业、生态农业、休闲农业，拓展农民增收空间。扎实推进“五大扶贫工程”，坚持扶贫攻坚与精准扶贫并重，着力构建专项扶贫、行业扶贫、社会扶贫三大格局，帮助3.84万农村贫困人口脱贫解困。

　　四、强化投资拉动，努力增强发展后劲

　　扩大投资规模。抓好256个市级重点项目和16个省重点项目建设，确保完成市级重点项目投资330亿元以上、省重点项目投资60亿元以上、全社会固定资产投资800亿元以上。健全项目全程服务机制，提高开工率、竣工率、投产率。加快构建“成渝立交桥”，完成内威荣高速公路（内江段）、自隆高速公路（内江段）主体工程建设，加快建设内江城市过境高速公路、黄荆坝大桥、内江公交枢纽中心站，开工建设内江综合客运中心，推动成渝客专按计划通车和川南城际铁路开工建设，加快推动向家坝灌区一期工程。加大土地整理和收储工作力度，推进工矿废弃地复垦利用工作，集约节约利用土地，确保近五年平均供地率高于60%。

　　优化投资结构。围绕产业发展、基础设施、生态环保、民生工程、社会事业等重点领域，抓好一批高端成长型产业、新兴先导型服务业项目，促进更多资金流向高新技术产业和战略性新兴产业。切实发挥政府性资金的导向作用，积极推进PPP等模式，鼓励民间资本参与重点项目建设，推动民间投资占全社会投资的比重稳步上升。

　　做好项目储备。把握国家和省投资政策取向，做好铁路、棚户区改造、城市基础设施、节能环保等领域项目的包装储备，完善重大项目库。加强项目规划、可研、选址、环评等前期工作，提高项目成熟度，力争更多项目列入全省50个重点推进项目和100个重点支持项目。

　　五、推进新型城镇化，充分释放发展潜力

　　健全城镇体系。完善城乡规划，推动水、电、路、气、通讯等基础设施和公共服务体系城乡联网共享、有效衔接，加快构建形态合理、功能完善、层级清晰的现代城镇体系，新增城镇建成区面积10平方公里以上，城镇化率提高1.6个百分点。加快建设内江新城，继续完善路网骨架，重点实施城市桥梁和次干道建设，启动321国道城区段提升改造、甜都大道北延线北段等道路建设。加快建设县城新区，确保县城控规覆盖率90%以上。支持两区改旧拓新，加快建设城南新区、白马组团和椑木组团。支持三县扩容提质，促进资中古城核心区与新城组团互动协调发展，助推隆昌加快构建城湖一体格局，深入推进威远北部新区建设。抓好11个全国重点镇和15个全省“百镇建设行动”试点镇建设，打造一批工业经济、商贸物流、现代农业、生态旅游强镇。

　　完善城市功能。以“两个加快”为着力点，推进新城核心区建设，启动农产品综合批发市场二期、五星小学、第三水厂、第二污水处理厂等项目，加快高铁站场、新城片区和高新区白马园区输电设施迁改等项目建设。加快实施“城市变景区”项目，启动新坝大桥至花园滩大桥滨河公园、牛毛山—五星水库主题公园等公园绿地项目，打造城区南北向、东西向两条生态廊道。围绕“把内江打造成为全省最整洁、最美丽的城市”目标，加强城市精细化管理，持续推进国家卫生城市创建，强力治理脏、乱、差；继续实施城中村、老旧院落、背街小巷改造提升工程，强力推进内棉片区、容器厂、滨江东路等棚户区改造；打好“交通治堵三年攻坚战”，优先发展公共交通，完成邱家嘴立交桥、桐梓坝大桥南成渝铁路桥下道路拓宽改造项目建设，启动建设沱江大桥复线桥，缓解城区交通压力，不断改善城市人居环境。

　　推进农业转移人口市民化。出台农业转移人口市民化实施办法，配套完善进城农民就业、社会保障等政策，健全城乡社会保险转移接续机制，扩大进城农民工住房保障范围。加强进城农民农村权益保障，探索进城落户农民自愿有偿退出或转让宅基地机制，保障农民宅基地用益物权，让农民愿进城、能进城、留得住。加大城乡统筹力度，促进更多公共资源和公共服务向农村延伸，逐步缩小城乡差距。

　　六、深化改革开放，激发发展动力活力

　　积极稳妥深化各项改革。继续推进农村产权制度改革，抓好市中区全国第二批农村改革试验区工作，全面完成确权颁证工作。深化行政审批制度改革，不断优化行政审批流程，加强行政服务体系建设。稳妥有序推进公务用车制度改革，创新公务出行保障方式。分类推进事业单位改革，深化教育卫生领域改革。加快国有资产改革，探索发展国有资本、集体资本、非公有资本交叉持股、相互融合的混合所有制经济。继续推进工商登记制度改革，激发民营经济活力。深化财政领域改革，提高依法理财水平。加快内江市农村商业银行组建和隆昌县农村信用社改制工作。

　　坚定不移扩大开放合作。继续打好“三大攻坚战”，全年到位市外资金465亿元。围绕高端成长型产业、新兴先导型服务业和传统优势产业，引进一批产业带动强、税收贡献大、就业效果好的优质项目，确保到位资金中制造业项目资金占比不低于50%。抢抓国家扩大内陆沿边开放和建设长江经济带、丝绸之路经济带等机遇，加快建设川渝合作“桥头堡”。加强厅市合作、校地合作、民间交流合作，促进互利发展。加快省级外贸发展示范基地建设，全力支持内江海关尽早开关，鼓励企业走出去，积极拓展海外市场。

　　七、发展社会事业，切实办好民生实事

　　扎实推进民生工程。继续实施“十项民生工程”，倾力办好民生实事，切实保障民生资金投入。优化就业创业服务，加快创建创业型城市，新增城镇就业3.65万人。扩大养老、医疗等社会保障覆盖面,推进城乡困难群众重特大医疗救助工作,启动市精神病康复中心建设、市第二社会福利院迁建等项目，建成县（区）标准化综合养老服务中心5个、城乡社区日间照料中心85个、老年活动中心82个。继续开展农村水井水质监测工作，扎实整改水质不合格水井。启动建设保障性住房900套，改造危旧房、棚户区11475户。在确保不欠新账的前提下，全面完成失地农民安置还房建设任务。强化价格监控，稳定物价水平。

　　加强生态环境保护。狠抓企业污染挂牌整治，加强环境风险隐患评估、排查、治理。鼓励开展节能减排技术开发与推广应用，大力推进工程减排、结构减排、管理减排，促进绿色低碳循环发展，完成“十二五”万元GDP综合能耗下降目标。深化工业污染治理，推进工业废水、废气、固体废弃物综合循环利用，全面淘汰10蒸吨以下小锅炉。加强畜禽养殖、农村面源污染治理，实施重点塘库“清水工程”。认真落实最严格水资源管理制度，加强甜城湖等饮用水源保护管理，推进市中区、资中县“节水型社会重点县”项目建设，促进威远河、隆昌河水质持续改善。强化空气质量监测，全面实施机动车尾气检测和环保标志管理，加大黄标车和老旧汽车淘汰力度，综合整治城市扬尘，改善空气质量。全力创建省级森林城市，城区新增绿地500公顷；争取纳入国家新一轮退耕还林工程，完成成片造林4万亩，确保森林覆盖率提高0.5个百分点以上。

　　办好人民满意教育。深入实施第二轮“学前教育三年行动计划”，新改扩建城乡公办幼儿园10所。建成义务教育标准化学校100所以上，着力化解城区“大班额”问题，确保威远县、隆昌县率先实现义务教育均衡发展。巩固高中教育质量，提升名校品牌影响力，抓好内江六中高新校区新建、内江一中扩建。大力发展职业教育，促进校企合作、产学对接，加快建设内江高级技工学校，推进内江师院整体迁建工程，积极创建四川健康职业学院。鼓励引导民间资金办学，提高民办教育质量。

　　提升卫生服务水平。加快市一医院、二医院、中医院新区建设。健全分级诊疗制度，力争县域内就诊率达90%。稳步提高新农合报销比例，减轻农村大病患者医药费用负担。开展爱国卫生运动，加强重大传染病防控，确保全市无重大传染病暴发流行。做好人口和计划生育工作，稳定低生育水平，提高人口质量。完善基层食品药品安全监管体系，严厉打击不法行为，保障食品药品安全。

　　繁荣文化体育事业。实施“十大社会事业惠民工程”，力争一批项目早日建成投用。推进文化惠民，深化“送文化下乡进社区活动”，完成“十二五”社区书屋建设任务。实施文化精品工程，加强非物质文化遗产保护，积极发展公共广播电视事业，弘扬优秀传统文化，引导人民群众践行社会主义核心价值观。深入开展全民健身运动，规划建设一批群众身边的文化体育设施。

　　积极推进军民融合深度发展，强化国防动员和后备力量建设,做好双拥共建、优抚安置和人民防空等工作。依法管理民族宗教事务,协调推动机构编制、统计、调查、新闻出版、无线电管理、外事侨务、检验检疫、防灾减灾、气象、水文、保密、档案、史志、老龄、残疾人、妇女儿童、红十字等事业发展。

　　八、加强社会治理，促进社会和谐稳定

　　创新社会治理。开展城乡社区标准化建设，推进网格化服务管理，推广村（居）民议事会，提升公共事务管理群众参与度。积极培育公益慈善组织，引导人民团体和社会组织完善法人治理结构，实现健康有序发展。全面推进社区矫正工作，加强社工人才和志愿者队伍建设，建立社区、社会组织、社会工作联动服务机制。

　　维护社会稳定。深化平安内江建设，完善立体化社会治安防控体系，防范、化解、管控影响社会安定的问题，依法打击各类违法犯罪活动。坚持用法治思维和法治方式化解社会矛盾，拓宽社情民意表达渠道，落实涉法涉诉信访依法终结制度，创新“大调解”工作体系，完善调解、仲裁、行政裁决、行政复议、诉讼等有机衔接的多元化纠纷解决机制。加强信息分析研判，做好舆情监测和引导工作。健全协调联动和快速反应机制，提高突发事件应急处置能力。

　　狠抓安全生产。严格落实安全生产责任制，加强煤矿、非煤矿山、危险化学品、交通运输、建筑施工、特种设备、消防等重点行业领域和人员密集场所的安全监管，保持“打非治违”高压态势。坚持重大隐患挂牌督办制度，实行分级分类监管，防范重特大生产安全事故发生。加大安全生产投入，深化科技兴安，推进安全生产标准化建设。做好地质灾害防治、防汛抗旱和森林防火工作。

　　九、加强自身建设，着力建设法治政府

　　严格依法行政。坚持崇尚法治、厉行法治，始终把法治放在心中、始终在法治轨道上运行，全力推进法治政府建设。坚持重大事项向人大及其常委会报告，规范性文件向人大常委会备案，主动接受人大及其常委会的法律监督、工作监督和政协的民主监督，认真办理人大代表建议、议案和政协委员提案。自觉接受司法监督，高度重视社会监督和舆论监督，广泛听取民主党派、工商联、无党派人士和工会、共青团、妇联等人民团体意见。严格遵循重大行政决策法定程序，不断完善重大决策法律咨询、法律支持、合法性审查、风险评估机制。推行权力清单、责任清单、负面清单制度，做到法定职责必须为、法无授权不可为，依法全面履行政府职能。加大普法工作力度，坚持政府常务会集中学法制度，深入开展“法律七进”活动，进一步加强法治文化建设。完善执法程序，推进综合执法，健全执法绩效考核和责任追究制度，确保公正文明执法。加强行政复议规范化建设，积极开展法治示范创建活动。

　　推进高效理政。继续整治“庸懒散浮拖”现象，坚决整治行政不作为、乱作为、慢作为等问题，深化党风政风行风群众满意度社会评价，不断提高行政效能和执行力。着力巩固党的群众路线教育实践活动成果，建立健全密切联系群众、改进工作作风常态化制度，深入基层、深入群众，敢于负责、勇于担当，提高工作效率和服务质量，让人民群众从政府工作和服务中感受到新风正气。推进依法理财，加强预算管理，强化财政（投资）评审工作，提高财政资金使用绩效，严控“三公”经费支出，推进节约型政府建设。依法规范和深化政务公开，推进公共资源配置、重大建设项目、公益事业建设等领域的政府信息公开。抓好行政权力清理、认定、公布工作，促进行政权力公开运行平台有效运行。

　　坚持廉洁从政。认真落实党风廉政建设责任制，加快建设廉洁政府。坚持用制度管权管事管人，强化廉政风险防控，完善公共资源交易监管制度，加强对项目招投标、土地使用权和矿业开采权出让、政府采购等重点领域和关键环节的监管。强化行政监察和审计监督，对公共资金、国有资产、国有资源、领导干部履行经济责任情况，实行审计监督全覆盖。严肃查办各类腐败案件和侵害群众利益案件。

　　2015年，在抓好以上工作的同时，我们将认真研究今后一段时期内江发展面临的新形势，主动适应转型升级、创新驱动的新要求，认真思考经济发展、社会进步的新举措，编制好“十三五”经济社会发展规划，确定今后五年发展的总体思路、目标任务、重点工作，引领内江未来发展。

　　各位代表，让我们紧密团结在以习近平同志为总书记的党中央周围，在省委、省政府和市委的坚强领导下，在市人大、市政协的监督支持下，紧紧依靠全市人民的智慧和力量，振奋精神、拼搏实干，抢抓机遇、乘势而进，为建设充满希望、充满活力的幸福美丽内江而努力奋斗！
